# Spice: discovery of phenotype-determining component interplays

**DOI:** 10.1186/1752-0509-6-40

**Published:** 2012-05-14

**Authors:** Zhengzhang Chen, Kanchana Padmanabhan, Andrea M Rocha, Yekaterina Shpanskaya, James R Mihelcic, Kathleen Scott, Nagiza F Samatova

**Affiliations:** 1 Department of Computer Science, North Carolina State University, Raleigh, NC 27695, USA; 2 Computer Science and Mathematics Division, Oak Ridge National Laboratory, Oak Ridge, TN 37831, USA; 3 Department of Civil and Environmental Engineering, University of South Florida, Tampa, FL 33620, USA; 4 Trinity College of Arts and Sciences, Duke University, Durham, NC 27708, USA; 5 Department of Integrative Biology, University of South Florida, Tampa, FL 33620, USA

## Abstract

**Background:**

A latent behavior of a biological cell is complex. Deriving the underlying simplicity, or the fundamental rules governing this behavior has been the Holy Grail of systems biology. Data-driven prediction of the system components and their component interplays that are responsible for the target system’s phenotype is a key and challenging step in this endeavor.

**Results:**

The proposed approach, which we call System Phenotype-related Interplaying Components Enumerator (Spice), iteratively enumerates statistically significant system components that are hypothesized (1) to play an important role in defining the specificity of the target system’s phenotype(s); (2) to exhibit a functionally coherent behavior, namely, act in a coordinated manner to perform the phenotype-specific function; and (3) to improve the predictive skill of the system’s phenotype(s) when used collectively in the ensemble of predictive models. Spice can be applied to both instance-based data and network-based data. When validated, Spice effectively identified system components related to three target phenotypes: biohydrogen production, motility, and cancer. Manual results curation agreed with the known phenotype-related system components reported in literature. Additionally, using the identified system components as discriminatory features improved the prediction accuracy by 10% on the phenotype-classification task when compared to a number of state-of-the-art methods applied to eight benchmark microarray data sets.

**Conclusion:**

We formulate a problem—enumeration of phenotype-determining system component interplays—and propose an effective methodology (Spice) to address this problem. Spice improved identification of cancer-related groups of genes from various microarray data sets and detected groups of genes associated with microbial biohydrogen production and motility, many of which were reported in literature. Spice also improved the predictive skill of the system’s phenotype determination compared to individual classifiers and/or other ensemble methods, such as bagging, boosting, random forest, nearest shrunken centroid, and random forest variable selection method.

## Background

Dynamic biological systems, such as cells, are inherently complex. This complexity arises from the selective and nonlinear interconnections of functionally diverse system components to produce coherent behavior. The key challenge is to reveal underlying simplicity from complexity [1]. Unlike the four Maxwell’s equations describing all the electro-magnetic phenomena from “first principles,” the fundamental rules that quantify the low dimensional behavior of biological systems are yet to be discovered.

Complementing approaches based on first principles, where the underlying system model is described by a system of equations, the data-driven modeling of system behavior is a promising approach. It aims to interrelate data from disparate and noisy experiments and observations to find informative features and link them to formulate fundamental principles governing a complex behavior. This process frequently begins with a comprehensive enumeration of the system “components” (e.g., co-regulated proteins in a cell) derived from experimental data. Discovery of putative associations between these “components” can then be used to design in silico system models (e.g., positive and negative feedbacks, information processing and signal transduction cascades) to better understand real system behavior.

To somewhat simplify this intricate process, data-driven characterization of a complex system behavior often starts with defining a target set of system’s distinct phenotypes of interest, such as thermo-resistance, acid-tolerance, hydrogen production, and enumerating only those key system components that could be responsible for or contributing to the given phenotype(s). For example, if the target phenotype is ethanol production by microbial cells via biomass degradation, then enumeration of phenotype-related system components would identify *all* the groups of proteins involved in degradation of cellulose to sugars, transport of these sugars through the membrane, and their fermentation to ethanol. Similarly, enumeration of *all* the cancer-related cellular components would identify all the genes that are likely related to the expression of cancerous cellular phenotype.

The difficulty in enumerating all the phenotype-related system components lies in dealing with the enormous number of system components (or features) that could easily reach thousands or even hundreds of thousands. Such enormous feature space could easily lead to the problem, coined by Bellman as “the curse of dimensionality” [2]. The problem gets complicated if one needs to select all those features that would provide clear differentiation between the true and merely feasible associations with the target phenotype. In addition, hierarchical nature of most biological systems leads to “short- and long-range” interactions between the features, or system. For example, hydrophobic residue pairs could enhance a propensity for other adjacent hydrophobic pairs (“short-range” feature correlation). On the other hand, highly specific residue interactions may be under selective pressure to fit into an overarching architectural motif (such as helix-turn-helix motif), thus contributing to “long-range” feature dependencies.

Moreover, it is often the case that a coordinated, not independent, action of several system components determines what phenotype(s) a given system will likely express. A system response represents a complex process, involving a series of (frequently induced) interacting events. Such non-linear cooperative or competing interactions between the system components often form hierarchical functional modules (e.g., communities) that act not only on different spatial and temporal scales but also in response to fluctuations induced by endogenous and exogenous factors. Hence, the approaches that identify individual components that confer a given system phenotype are likely not optimized to detect groups of such interplays between system components. Instead, there is a need for methods that aim to enumerate all the groups of *cross-talking* system components that could be associated with the system phenotypic state. We call this problem the enumeration of system phenotype-determining component interplays.

To address this problem, we propose an iterative, classification-driven approach that comprehensively enumerates the set of feature subsets that discriminate between different system phenotypes (or classes). We define a system component (a protein or group of proteins) as a feature in this paper. Given a set of observations about system components (features) with the corresponding assignment of the system’s phenotype (class), our method measures the importance of feature subsets to discriminate between system phenotypes. Despite combinatorial complexity of the problem, our method almost exhaustively explores feature subsets based on information-theoretic selection and dense enriched subgraph enumeration process. Our method rests on a hypothesis that if a subset of system components discriminates between system’s functional states, then when considered altogether, these components most likely form a cross-talking phenotype-determining feature subset. It also places the contribution of an entire feature subset at the core of the analysis as opposed to the approaches that first evaluate the importance of individual features and then filters those that are associated with a particular system’s phenotype. It further filters those feature subsets that are statistically significant, and are thus assumed to be relevant to the target phenotype(s). Our method can be applied to both instance-based data such as microarray patient sample data and network-based data such as gene networks.

The major contributions of this work are as follows: 

1. We propose an algorithm, named Spice, to address the new problem of enumeration of system phenotype-determining component interplays. Spice iteratively enumerates all the groups of statistically significant *cross-talking* system components, which, to the best of our knowledge, no existing methodologies are particularly designed for.

2. We evaluate our method on both instance-based data and network-based data to identify system components related to three target phenotypes: biohydrogen production, motility, and cancer. We show that the identified phenotype-related components are biologically relevant and consistent with the results in literature.

3. Additionally, we apply our method to eight benchmark microarray data sets to show its effectiveness and robustness on the phenotype-classification task.

### Related work

To the best of our knowledge, the proposed problem of enumerating statistically significant component interplays that are key contributors to the system’s phenotype has not been addressed in literature. The problem resembles, yet with quite apparent distinctions, the problems of feature selection, phylogenetic profiling, network alignment, and frequent subgraph mining.

At a higher level, these problems could be divided into two major categories depending on whether pairwise relationships between system components are known. If they are defined, then the system could be modeled as a complex network, and multiple network alignment approaches [3,4] that look for subgraphs that co-occur across multiple network instances for the same system’s phenotype are putative candidates for the target component interplays. The key limitation of this strategy is that such approaches aim to identify the component groups that are present in all or most of a given set of network instances and would likely miss those that are only common to a subset of the instances. Likewise, they are not equipped with any means to suggest that these groups are specific to the target system phenotype and not common to multiple system phenotypes. While the former limitation is addressed by the approaches based on frequent subgraph mining [5,6], similar comments would still hold for the latter comment. In addition, the runtime for these approaches grows exponentially; even the most efficient ones, such as MULE [5] that enumerates maximal frequent edge sets, took almost 57 days for a set of 98 network instances (details available upon request). While efficient heuristics have been reported [7], they are tailored for specific network types (e.g., metabolic networks).

For the second category, the system is often represented by its set of components (i.e., features) that are defined over multiple instances (i.e., observations) for each of the finite set of system’s distinct phenotypes. In this case, univariate approaches, such as those that, for the given feature, look for a strong correlation between its profile and the system’s phenotype profile across multiple instances identify a set of putative candidates for component interplays. Different correlation measures, such as Pearson correlation, Mutual Information, Student’s *t*-test, ANOVA, Wilcoxon rank sum, Rank products, and other univariate filter feature selection techniques can provide different candidate sets that could be further assessed with set-theoretical approaches to provide either higher specificity (i.e., intersection of sets) or higher sensitivity (i.e., set union).

A particular instance of such a strategy is phylogenetic profiling [8], where different organisms that exhibit various (but finite) phenotypes (e.g., aerobic vs. anaerobic growth) are considered as observations characterized by the the presence or absence of particular genes (or components). The underlying hypothesis behind this approach is that candidate genes are more likely to be present in phenotype-expressing organisms than in phenotype-non-expressing organisms due to an evolutionary pressure to conserve the phenotype-related genes [9]. While simple, fast, and effective [10] in finding individual components that are likely associated with the system’s phenotype, such methods are quite limited in discovering of the component interplays.

Multivariate feature selection approaches could be considered as the closest approximation to the proposed problem. The multivariate feature selection approaches can be broadly divided into the following categories: (1) filter techniques (e.g., fast correlation-based algorithm [11]), (2) wrapper techniques (e.g., GA/KNN method (combining a Genetic Algorithm (GA) and the k-Nearest Neighbor (KNN) method) [12]), and (3) embedded techniques (e.g., random forest [13]). In filter techniques, the relevance of features is evaluated according to some metric, and the features with the top *k* ranking are then selected for further analysis. Filter feature selection techniques are simple, fast, and effective, but these techniques often ignore the correlations between different features. In biology, these correlations depict protein interactions and should not be ignored. Wrapper methods take the dependencies between the features into account, but suffer from overfitting problem. Additionally, they are often computationally expensive. Embedded methods can be far less computationally expensive than wrapper methods, but these approaches are very specific to a given classification algorithm.

Our work is also related to network-based identification methods. Network-based identification methods aim to incorporate pathway or gene network information (typically generated from expression datasets) information to help identify functional modules, or improve the prediction. Pathway-based methods [14,15] try to detect the network pathways by assuming that the genes inside a module are co-expressed. However, pathway-based methods ignore the detailed network topology, and a small perturbation that is likely to affect many “modules” [16]. While integrating of gene expression information into identification of gene modules is biologically meaningful, gene-network based methods are rarely satisfactory because they either focus on small networks by using the greedy subgraph search algorithm [17,18] or focus on detecting non-overlapping subnetworks [16,19,20].

## Results and discussion

The nature of the proposed methodology, System Phenotype-related Interplaying Components Enumerator (Spice) (see Method section), suggests that detected component interplays (Steps 1-4) (1) could play an important role in defining the specificity of the system’s phenotype(s); (2) would likely exhibit stronger inter-component relationships within the same group than between the groups and are functionally coherent, likely, act in a coordinated manner to perform the phenotype-specific function; and (3) collectively, could improve the predictive skill of the system’s phenotypes (Step 5).

### Phenotype-specificity determining components

#### Groups of enzymes associated with biohydrogen production

Biological hydrogen is a promising renewable energy source [21], which can be generated by utilizing one of three metabolic processes: light fermentation, dark fermentation, or photosynthesis [22]. To date, a number of phylogenetically diverse microorganisms have been identified as hydrogen producing. Such organisms include photosynthetic bacteria, nitrogen-fixers, and heterotrophic microorganisms [23]. In order to generate hydrogen, these organisms may rely upon one or more metabolic routes. As such, the biohydrogen production phenotype provides an opportunity to evaluate the capabilities of Spice to handle a relatively complex phenotype. Identification of phenotype-related components was based on the assumption that if a component (i.e., a group of enzymes in a metabolic process) is specific to biohydrogen production, then it is likely evolutionarily conserved across *H*_2_-producing organisms, and it is absent in most *H*_2_-non-producing ones.

Our first experiment includes the data about 17 *H*_2_-producing and 11 *H*_2_-non-producing microorganisms (see Additional file 1) and compares Spice’s performance against the two commonly used statistical methods: Mutual Information (MI) and Student’s *t*-test, and one multivariate feature selection approach: SVM recursive feature elimination (SVM-RFE). Among 17 *H*_2_-producing microorganisms, four microorganisms utilize bio-photolysis, five microorganisms utilize light fermentation, and eight microorganisms utilize dark fermentation. 11 microorganisms are listed as non-hydrogen producing because they are not associated with hydrogen production based on literature review, or they lack hydrogenase [24], one of the key enzymes involved in hydrogen production. All microorganisms used in this experiment were verified as completely sequenced using the NCBI database. The input to SPICE is a matrix, with the enzyme EC numbers along the rows, 28 organisms (hydrogen producing and non-producing) along the columns, and the entry in each cell (*i, j*) is the copy number for enzyme *i* in organism *j*. The last row of the matrix includes information about the organism’s ability to express the hydrogen production phenotype.

The mutual information method [25] assesses correlation between the enzyme’s phylogenetic profile and the organism’s *H*_2_-production profile across multiple organisms. In addition, it reports a significance threshold by shuffling the enzyme profile vectors and calculating the mutual information with the organism’s phenotype profile. Only those enzymes, whose mutual information values lie above the confidence cutoff are reported.

The Student’s *t*-test is another statistical method to identify phenotype related enzymes, where we utilize the enzyme phylogenetic profiles alone to measures statistical bias of enzyme copy numbers in one phenotypic group of organisms vs. the other. The test results are filtered so that only enzymes with the *p*-value less than 0.05 are considered significant.

Guyon *et al.*[26] proposed the SVM-RFE algorithm to rank the features (enzymes) based on the value of the decision hyperplane given by the SVM. The features with small ranking scores are removed. The top 240 enzymes (out of 1,229 enzymes) are considered significant.

**Figure 1:**
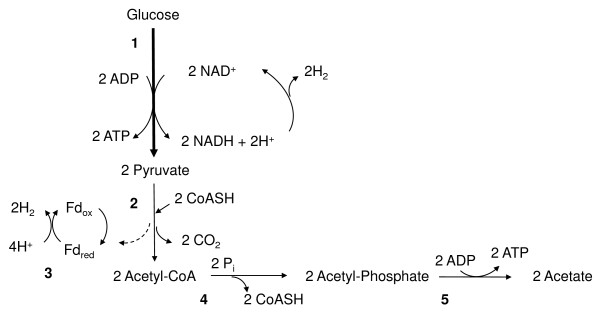
**Fermentation of glucose to generate acetate.** Schematic of key metabolic pathways for hydrogen production in *Clostridium acetobutylicum*. Arrows with larger width indicate a series of reactions. Arrows with narrow width indicate individual reactions. Enzymes: 1, glycolytic enzymes; 2, pyruvate ferredoxin oxidoreductase (E.C. 1.2.7.1); 3, hydrogenase (E.C.1.12.7.2); 4, phosphotransacetylase (E.C. 2.3.1.8); 5, acetate kinase (E.C. 2.7.2.1)

**Figure 2:**
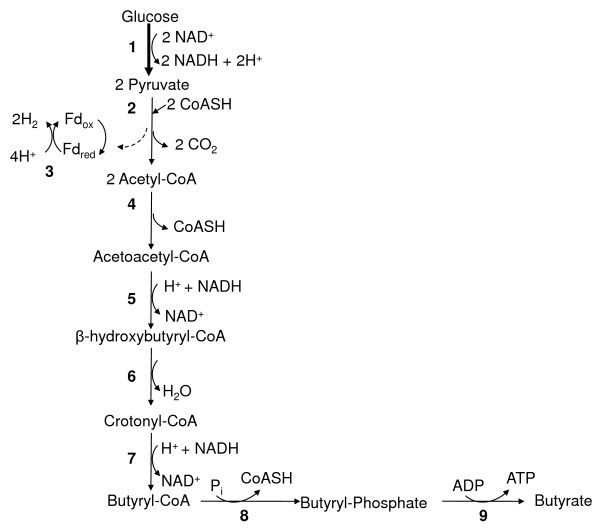
**Fermentation of glucose to generate butyrate.** Schematic of key metabolic pathways for hydrogen production in *Clostridium acetobutylicum*. Arrows with larger width indicate a series of reactions. Arrows with narrow width indicate individual reactions. Enzymes: 1, glycolytic enzymes; 2, pyruvate ferredoxin oxidoreductase (E.C. 1.2.7.1); 3, hydrogenase (E.C.1.12.7.2); 4, acetyl-CoA acetyltransferase (thiolase) (E.C. 2.3.1.9); 5, *β*-hydroxybutyryl-CoA dehydrogenase (E.C. 1.1.1.157); 6, crotonase (E.C. 4.2.1.55); 7, butyryl-CoA dehydrogenase (E.C. 1.3.99.2); 8, phosphotransbutyrylase (E.C.2.3.1.19); 9, butyrate kinase (E.C. 2.7.2.7). Abbreviations: Ferredoxin (Fd); Coenzyme A (CoASH).

Figure 1 and Figure 2 show the pathway and key enzymes for hydrogen production from the fermentation of glucose to acetate (Figure 1) and butyrate (Figure 2) in *Clostridium acetobutylicum*. Within this process, glucose is broken down through a series of glycolytic enzymes to generate pyruvate. Pyruvate is then converted to acetyl-CoA through the action of pyruvate ferredoxin oxidoreductase. During this step, hydrogen gas is produced when pyruvate is oxidized, thus resulting in the formation of *CO*_2_ plus *H*_2_. Production of hydrogen via this route is mediated through two enzymes—pyruvate ferredoxin oxidoreductase and hydrogenase. Acetyl-CoA generated produced from pyruvate can then enter a number of pathways, including the acetate and butyrate formation pathways.

While production of hydrogen occurs predominately during formation of Acetyl-CoA and not in the secondary pathway (e.g., conversion of Acetyl-CoA to acetate), acetate and butyrate fermentation pathways play an important role in the overall yield of hydrogen by microorganisms. In metabolic engineering studies, the goal is to generate the highest theoretical yield of hydrogen through alteration of metabolic routes or key enzymes related to hydrogen production.

For enhanced hydrogen production, acetate is the desired end product because of its higher hydrogen yield compared to other by-products, such as butyrate [27,28]. Specific differences in conversion efficiencies can be observed by comparing the two chemical reactions below:

(1) $$\begin{array}{ll} C_{6}H_{12}O_{6} & +2H_{2}O\to 2{\mathrm{CH}}_{3}\text{COOH}\\ & +2CO_{2}+4H_{2}:\text{glucose into acetate} \end{array}$$

(2) $$\begin{array}{ll} C_{6}H_{12}O_{6} & \to CH_{3}CH_{2}CH_{2}\text{COOH}\\ & +2CO_{2}+2H_{2}:\text{glucose into butyrate} \end{array}$$

The first reaction shows that the maximum theoretical hydrogen yield is 4 *H*_2_ per mol of glucose produced when acetate is the end product [29,30], compared to a maximum theoretical hydrogen yield of 2 *H*_2_ with butyrate as the end product [27,31,32]. During acetate and butyrate formation, 2 mols of hydrogen are generated during reaction 3 when pyruvate ferredoxin oxidoreductase reduces ferredoxin (Fd) and hydrogenase immediately oxidizes it to generate *H*_2_ (Figures 1 and 2). When acetate is the only end product as depicted in Figure 1, then additional hydrogen is produced when *2NAD*^+^ is reduced to form *2NADH + 2H*^+^ (reaction 3). An illustration of the two reactions is shown in Figure 1 (acetate) and Figure 2 (butyrate).

Due to the importance of acetate and butyrate production in the generation of hydrogen production, we evaluated the ability of Spice to identify these two pathways. Results show that Spice identified all of the acetate pathway’s constituent enzymes, including acetate kinase (E.C. 2.7.2.1), as being significant. In contrast, the Student’s t-test and the MI method did not find any of the enzymes, and SVM-RFE detected acetate kinase. Additionally, all five enzymes active in the butyrate pathway [28] were found by the Spice method. Among these, only three were discovered by the SVM-RFE, two were found by the Student’s t-test and none by the MI method.

Within facultative anaerobes like *Escherichia coli*, hydrogen gas may be produced directly through the production of formate. In this pathway, pyruvate is converted to formate and acetyl-CoA with the use of pyruvate formate lyase (E.C. 2.3.1.54) [33]. The formate hydrogen lyase complex made up of formate dehydrogenase and ferredoxin hydrogenase breaks down the formate into hydrogen gas and carbon dioxide [28]. In this study, pyruvate formate lyase was found by the Spice method to be significant.

Table 1 shows that Spice detected all the enzymes (see Additional file 2) specific to the three pathways in facultative anaerobes, such as *Escherichia coli*, while mutual information could not even discover a single enzyme, Student’s t-test could only detect 2 enzymes, and SVM-RFE could find four out of 7 enzymes. Thus, Spice outperformed, in terms of sensitivity, the existing state-of-the-art methods based on Student’s t-test, MI, and SVM-RFE. The enzymes identified by Spice are next described in the context of their corresponding metabolic pathways.

**Table 1:** ***H***_***2 ***_**-related enzymes detected by different methods**

**Pathway**	**Enzyme**	**Enzyme Name**	***t***	***MI***	**SVM-RFE**	**Spice**
Acetate	2.7.2.1	acetate kinase			** + **	** + **
	1.3.99.2	butyryl-CoA dehydrogenase			** + **	** + **
	2.7.2.7	butyrate kinase	** + **		** + **	** + **
Butyrate	1.1.1.157	3-hydroxybutyryl-CoA dehydrogenase				** + **
	2.3.1.19	phosphate butyryltransferase	** + **			** + **
	2.3.1.9	acetyl-CoA C-acetyl-transferase			** + **	** + **
Formate	2.3.1.54	pyruvate formate lyase				** + **

Note: *t*: Students’ *t*-test; *MI*: Mutual Information.

#### COG modules corresponding to biohydrogen production

To expand our study beyond metabolic subsystems to include possible regulators, transporters, and others, in our next experiment, we replace enzymes in the matrix with the clusters of orthologous groups (COGs) [34]. We obtain COG–organism association information from the STRING database. The new COG-centric matrix for this experiment can be found in Additional file 3.

The set of enumerated COG modules with the statistically significant *p*-value of 0.05 is provided in Additional file 4. Spice was able to identify COG modules that are known to be associated with hydrogen production based on our literature review and prior knowledge. Next, we will briefly summarize some of these modules.

##### COG modules related to nitrogenase

In addition to the metabolic pathways described above, other key enzymes are known to be associated with hydrogen production in a number of microorganisms [35-37]. Examples of such enzymes include nitrogenase and hydrogenase enzyme complexes. Hydrogen producing organisms capable of fixing nitrogen contain enzyme complexes, termed nitrogenases. Within nitrogenase complexes, nitrogen gas is converted to ammonia, inadvertently resulting in the production of hydrogen gas as a byproduct [23,36].

Evaluation of the COG modules generated by Spice indicated the presence of two modules, each containing an essential component of enzyme complex nitrogenase. In the first module, two COGs (COG2710 and COG0120) were identified. COG2710 is associated with expression of the molybdenum–iron protein (NifD) [23] and COG0120 is associated with the protein—Ribose 5-phosphate isomerase (RpiA). NifD protein is one essential component of nitrogenase, serving as the binding site for substrates during nitrogen-fixation [23,38]. RpiA takes a vital part in carbohydrate anabolism and catabolism through its participation in the Pentose Phosphate Pathway (PPP) and Calvin Cycle [39]. In addition, studies of central metabolism indicate that RpiA is a protein highly conserved across many microorganisms [39]. However, in this study, RpiA was paired with NifD, suggesting that both proteins may be associated with nitrogen-fixation, hence biological hydrogen production. In terms of hydrogen production, metabolism of and the ability to metabolize specific carbohydrates play an indirect role in the over-production of hydrogen. One example is the *C. butyricum*. Metabolic studies of the *C. butyricum* demonstrate the ability of this bacterium to digest a variety of carbohydrates and to produce hydrogen via degradation of carbohydrates [40].

Another role RpiA may play is the production of NADPH required for fixing nitrogen [41]. In nitrogen fixers, the oxidative pentose phosphate cycle has been reported as active. During oxidative PPP, Riboluse-5-phosphate is converted to ribose-5-phosphate by Rpi. During this reaction, NADPH is generated, thus allowing for N assimilation, N-fixation, and production of hydrogen.

The second nitrogenase-related module identified by Spice contains COG1348 (NifH) and COG3883 (Uncharacterized). Similar to NifD, NifH is also considered to be an essential component of nitrogenase. It is responsible for assisting with the biosynthesis of co-factors for NifD [42]. COG3883 is uncharacterized. While we cannot predict the role of the protein from this module, its presence suggests that it is either associated with the nitrogen fixation or hydrogen production phenotype.

##### COG modules corresponding to hydrogenase

Hydrogenase enzyme complexes are key enzymes involved in the uptake and production of biological hydrogen [35]. Analysis of hydrogenase enzymes have identified three different types, each associated with a number of accessory proteins necessary for activation [35,43]. These include the [NiFe]-hydrogenase, [FeFe]-hydrogenase, and non-metal containing hydrogenase enzyme [35]. Due to the importance of hydrogenase in both hydrogen production and hydrogen uptake, several studies have examined the role of hydrogenase enzymes in a number of different hydrogen-producing organisms [44,45]. These studies have found many microorganisms, including *Clostridium acetobutylicum*, capable of having both hydrogen uptake (e.g., [FeFe]-hydrogenase) and hydrogen evolving enzymes (e.g., [NiFe]-hydrogenase). In this study, Spice predicted the presence of both hydrogen uptake and hydrogen evolving enzymes as related to the hydrogen production phenotype. Categorization of hydrogen uptake hydrogenases may be due to the absence of hydrogenase in microorganisms present in our data set.

In this study, Spice identified one module containing a hydrogen evolving hydrogenase. Within this module two COGs, COG4624 (iron only hydrogenase) and COG3541 (predicted nucleotidyltransferase) were present. The protein ID for COG4624 was not identified in the literature review; however, [Fe]-hydrogenases are responsible for producing hydrogen [46]. Nucleotidyltransferases are proteins involved in a number of biological processes ranging from DNA repair to transcription [47]. Since these proteins are generally involved in DNA and RNA-related processes, it is unclear why a predicted nucleotidyltransferase was paired with hydrogenase. To understand the interaction between these two proteins, experimental molecular analysis is necessary.

Another COG module found by Spice contains COG0068 and COG0025, which are associated with expression of two hydrogenase uptake proteins—hydrogenase maturation factor (HypF) and NhaP-type Na+/H+ and K+/H+ antiporters (Nhap). HypF has been found as a carbamoyl phosphate converting enzyme (or an auxiliary protein) involved in the synthesis of active [NiFe]–hydrogenases in *Escherichia coli* and other bacteria [48]. NT01CX_0020, an orthologous group of COG0025, is associated with expression of sodium/hydrogen exchanger protein (NHE3). NHE3 has been found to play an important role in hydrogen production of *Acidaminococcus fermentans*, *Escherichia coli* and bacterial communities within a dark fermentation fluidised-bed bioreactor [49-51].

Spice also identified three other types of hydrogenase maturation proteins—HypC, HypD, and HypE. COGs corresponding to these proteins are COG0298 (HypC), COG0409 (HypD), and COG0309 (HypE). Understanding complexes, such as uptake hydrogenase enzymes, is important for deciphering regulatory mechanisms and activity of these key enzymes. For example, in studies evaluating accessory proteins present in [NiFe]-hydrogenase complexes, HypCDEF proteins are described as regulators for maturation of uptake hydrogenase through participation in development of the active center [35,52]. If one of the Hyp proteins is missing, the entire complex is inactivated.

In *H*_2_–producing microorganisms such as *Escherichia coli*, hydrogenase maturation proteins act as regulators for maturation of uptake hydrogenase in development of the active center [35,36]. Regulation is conducted by inserting Fe, Ni, and diatomic ligands of HypA–F proteins into the hydrogenase center for activation and maturation [53]. To carry out this process, HypE and HypF are in charge of synthesis and insertion of Fe cyanide ligands into the hydrogenase’s metal center, and HypC and HypD are responsible for construction of the cyanide ligands [36,54].

In addition, Spice identified two hydrogenase proteins associated with anaerobiosis [55]. They are COG0374 (HyaB) and COG0680 (HyaD). Unlike the Hyp proteins, which are accessory proteins involved in the assembly of the metallocenters, Hya proteins are responsible for the maturation of hydrogenase-1 [46].

##### Other COG modules related to biohydrogen

Other biohydrogen production-related COGs, such as COG0374, COG0375, COG3261, COG0680, COG4624 and others, shown under the hydrogenase category in STRING database are detected as part of other modules by Spice. As mentioned earlier, hydrogenase is one of the key proteins (or enzymes) involved in hydrogen production and uptake [24]. The complete list of all the identified putative biohydrogen-related COG modules is available in Additional file 4.

#### Motility-related COG modules

For a large-scale experiment, we set up another experiment on a different phenotype—motility. A total of 141 organisms including 56 non-motile organisms and 85 motile organisms were chosen from Slonim *et al.*[8]. For *p*-value of less than 0.01, Spice detected 96 modules. The input data and results can be found in Additional files 5 and 6, respectively.

One of the motility phenotype-related COG modules contained COG1338, COG0265, COG1484, and COG3420. Among the four COGs, COG1338, whose function is associate with the expression of flagellar biosynthetic protein (Flip), has a high correlation with flagellar assembly pathway [56]. Flagellar assembly pathway, which enables the movement of microorganisms, is well-known to be important for bacterial motility [56,57]. Proteins associated with the other three COGs include uncharacterized serine protease (YyxA) and two hypothetical proteins. YyxA in a motile organism, *Bacillus amyloliquefaciens*, has a similar phylogenetic profile to chemotaxis-related proteins [58]. Chemotaxis pathway, which is also important for bacterial motility, determines how the microorganism moves according to its environment [8]. Chemotaxis pathway and flagellar assembly pathway function together to guide bacteria’s direction of movement [8]. The phylogenetic profile of the other two hypothetical proteins (associate with COG1484 and COG3420) are shown to be correlated with the pattern of motility across many bacterial genomes [8].

Additionally, Spice enumerated other COG modules that contained other known flagellar-related COGs like COG1516, COG1345, and COG1815 and other known chemotaxis-related COGs such as COG0840, COG0643, and COG0835, supported by literature [8,56,57]. Besides flagellar-related and chemotaxis-related COGs, type III secretion system-related COGs, such as COG1766, COG1684, COG1987, and COG1338, were also found in some of our enumerated modules. The type III secretion system is found to be highly correlated with bacterial motility, because some of its protein structure is very similar in structure, function, and gene sequence to the flagellar assembly system [56,59].

#### Cancer-related genes

Identifying *all* the genes that could discriminate tumor cells from normal cells in microarray gene expression data is non-trivial [60]. Again, the task is *not* to find a *single* “best”-discriminating gene set, but enumerate as many cancer-related genes and groups of genes as possible provided they are associated with cancer expression phenotype; this task is becoming particularly important in the context of personalized medicine.

Leukemia cancer data was selected to show the effectiveness of our method to detect phenotype-related gene modules in biological networks. Leukemia data can be downloaded from Broad Institute Cancer Program Data ( http://www.broadinstitute.org/cgi-bin/cancer/datasets.cgi). It contains 72 measurements for the expression of 7,129 genes, corresponding to the samples taken from bone marrow and peripheral blood. Out of these samples, 47 samples are classified as ALL (Acute Lymphoblastic Leukemia), and 25 samples are classified as AML (Acute Myeloid Leukemia).

The first 11 genes identified by Spice were used as seed set, and a total of 145 phenotype-associated gene functional modules (see Additional file 7) were generated by DENSE algorithm in the Leukemia network. 5 out of the 11 seed genes are filtered out by our method. Table 2 shows the first 5 models identified by our algorithm. Specifically, gene *KIAA0016* found by our model 1 is highly correlated with anti-cancer agents [61]. *KIAA0016* encodes TOMM20—a mitochondrial import receptor [62]. TOMM20 has been shown to interact with a central anti-apoptotic Bcl-2 (B-cell lymphoma 2) gene [63]. The expression of Bcl-2 has been used as a prognostic marker for acute myeloid leukemia [64]. *KIAA0035*, *Cellular nucleic acid binding protein* and *KIAA0016* belonged to a functional module in the Leukemia network. Our method also detected an overlapping functional module with only one gene (KIAA0242) difference to model 1. *Zyxin* found by our model 3 plays a vital role in mitosis [65], and the LIM Domain of *Zyxin* is known to interact with leukemogenic bHLH proteins, such as TAL1, TAL2, and LYL1 [66].

**Table 2:** **Cancer-related genes found bySpiceon Leukemia network**

**Model ID**	**Gene ID**	**Gene description**
	210	KIAA0016
Model 1	284	KIAA0035
	6889	Cellular nucleic acid binding protein
	210	KIAA0016
Model 2	284	KIAA0035
	744	KIAA0242
	4847	Zyxin
Model 3	4229	SPI1 Spleen focus forming virus
		1882	CST3 Cystatin C
Model 4	630	FCN1 Ficolin	
	1157	PI Protease inhibitor 1	
	1882	CST3 Cystatin C	
Model 5	5956	PSAP Sulfated glycoprotein 1	

Note: More cancer-related genes are found by other models.

### Predictive skill

#### Data

Eight publicly available multi-phenotype-genotype datasets are used in this study. Table 3 summarizes some characteristics of these datasets, their sources, and the best-to-date performance reported in literature. For comparison purposes, the last column indicates Spice’s performance.

**Table 3:** Performance comparison on microarray data sets

**Dataset**	**Features**	**Samples**	**Classes**	**Source**	**CV**	**Acc.**^***r***^**(%)**	**Acc.**^***♭***^**(%)**	Spice**(%)**
Leukemia	7129	72	2	[60]	10-fold	91.2	97.14 [67]	98.6
Colon cancer	2000	62	2	[68]	2:1 RP	87.14	87 [69]	89
B-cell lymphoma	4026	96	2	[70]	5:3 RP	92.1	93.55 [71]	94.7
Prostate	6033	102	2	[60]	10-fold	73.5	87 [72]	93.1
Lymphoma_3class	4026	62	3	[68]	2:1 RP	99.05	97.36 [73]	100
SRBCT	2308	63	4	[68]	2:1 RP	98.7	98.7 [69]	98.7
CNS^∗^	74	60	2	[60]	10-fold	88.3	75 [74]	96.7
Prostate outcome^∗^	208	21	2	[60]	10-fold	85.7	90 [75]	100

Notes: ^*^: Discretized data; CV: Cross-validation; RP: Random partition; ^r^: Accuracy from source reference; ^*♭*^: Accuracy reported in a recent literature.

#### Evaluation methodology

For two-class, 10-fold cross-validation are employed. 10-fold cross validation has been proved by Witten and Frank [76] to be a good way to evaluate the performance of a classifier. In 10-fold cross-validation, the original data is partitioned into 10 different subsets. Each of the 10 subsets is used as the test set, and nine other subsets are used as training set. For multi-class datasets, 3-fold cross validation is used to ensure that each subset can have all different classes of samples.

Bootstrapping validation, via commonly used bootstrap estimators, e0 bootstrap and .632 bootstrap [77], is also applied. In e0 bootstrap, the training data consists of n instances by re-sampling with replacement from the original data of the same size of *n*. And the test data is the set difference between original data and training data. Thus, if the training data has *j* unique instances, then the test data will be the other *n-j* instances on the original data. The error rate on the test data is treated as the e0 estimator, while the .632 bootstrap also takes the training error into consideration, and uses the linear combination of 0.368∗*ε* + 0.632∗*e*0 as the estimated error rate, where *ε* is the training error. For good error estimation, we use ≈200 iterations [77] and report the average error rate.

Bagging [78], boosting [79], random forest [80], nearest shrunken centroid method (PAM) [81], and random forest variable selection (varSelRF) [82] ensemble learning techniques are employed as benchmark methods. The ensemble size used for these methods is the same as the one used for Spice.

We utilize different skill metrics including accuracy, sensitivity, specificity, precision, *F*_1_-measure, variance, Heidke Skill Score (HSS) [83], Peirce Skill Score (PSS) [83], and Gerrity Skill Score (GSS) [83]. Accuracy is defined as the ratio of the number of correctly classified data points to the total number of data points in the test set. The HSS measures how well a forecast did as to a randomly selected forecast. PSS, also called “true skill statistic,” is another popularly skill score computed by the difference between the hit rate and the false alarm rate. GSS, also known as “threat” score or critical success index, is a particular useful measure of skill for situations where the occurrences of the event to be forecast are substantially less frequent than the non-occurrences [83].

#### Skill metrics evaluation

**Figure 3:**
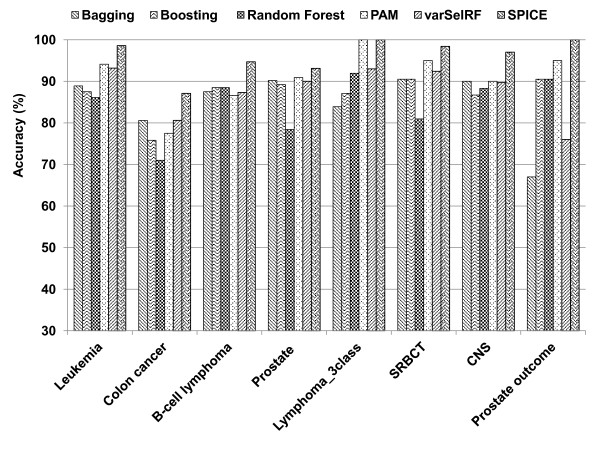
**Comparison of prediction accuracy of Spice to other ensemble classifiers on eight microarray datasets.**

Figure 3 shows cross validation accuracy of Spice compared to bagging, boosting, random forest, PAM, and varSelRF ensemble methods. We report the accurate results of bagging, boosting, random forest, PAM, and varSelRF by using the default parameters. CART decision tree is used as the base classifier for bagging, boosting, and Spice. To be consistent, we use 11 iterations as the stopping criterion (or the maximum ensemble size) for all the methods. Spice outperforms bagging, boosting, random forest, PAM and varSelRF by up to 33%, 13%, 18%, 10%, and 24%, respectively.

Table 4 summarizes Spice’s skill on two-class microarray data using five metrics: accuracy and its variance, sensitivity, specificity, precision, and *F*_1_-measure; it also reports an average number of features per model. Table 5 summarizes Spice’s skill on multi-class microarray data using five metrics: accuracy and its variance, HSS, PSS, and GSS.

**Table 4:** **Spiceperformance on two-class microarray data sets**

**Metric**	**Leukemia**	**Colon**	**B-cell lymphoma**	**Prostate**
Accuracy	0.99	0.87	0.95	0.93
Variance	0.001	0.001	0.000	0.000
Sensitivity	0.98	0.90	1	0.9
Specificity	1	0.82	0.85	0.96
Precision	1	0.90	0.92	0.95
_*F*1_-measure	0.99	0.90	0.96	0.93
Features	2.23	2.61	2.52	3.33

**Table 5:** **Spiceperformance on multi-class microarray data sets**

**Metric**	**Lymphoma_3class**	**SRBCT**
Accuracy	1.0	0.98
Variance	0.000	0.005
HSS	1	0.98
PSS	1	0.981
GSS	1	0.98

#### Different weighting schemes’ test

One factor that may influence the results of Spice method is the weights assigned to different candidate classifiers in the ensemble for determining the phenotype. Here, we test three different weighting schemes described in Step 5: bringing component interplays altogether section: majority voting, training accuracy-based voting, and internal cross-validation-based voting. The experimental results show that there is no bearing on prediction accuracy by choosing different weighting schemes for a majority of microarray datasets, although the training accuracy-based voting and internal cross-validation-based voting performed slightly better (3–5%) than the majority voting scheme on few datasets like the B-cell lymphoma dataset. However, all weighting schemes highly outperformed any single classifier in the ensemble.

#### Robustness assessment

To assess robustness, we applied bootstrapping using both e0 and .632 bootstrap estimators with 200 bootstrapping trials. Bootstrapping is applied to all three categories of data sets. Leukemia data is the original 2-class data without any preprocessing, CNS data is the discretized data, and Lymphoma_3class data is multi-class data with logarithmic transformation and standardization. Table 6 shows that Spice provides bootstrap error rates comparable with cross-validation results.

**Table 6:** **Bootstrapping performance ofSpice**

**Data**	**e0**	***ε***	**.632**	**10-fold cross validation**
Leukemia	0.037	0	0.024	0.014
CNS	0.044	0.031	0.007	0.030
Lymphoma_3class	0.027	0	0.017	0.000

#### Ensemble statistics

**Figure 4:**
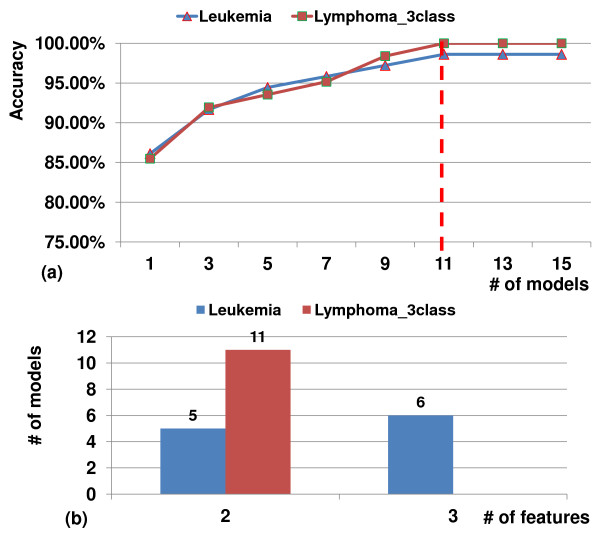
Ensemble statistics.

Figure 4 shows the ensembles built by Spice on Leukemia and Lymphoma_3class data, using 11 or fewer classifier models (Figure 4(a)), with each model including 2–3 features (Figure 4(b)). The fact that the ensemble uses information from multiple diverse models and achieves a good accuracy with only a few features per model is a good indicator for our classifier ensemble methodology.

#### Algorithm efficiency

**Figure 5:**
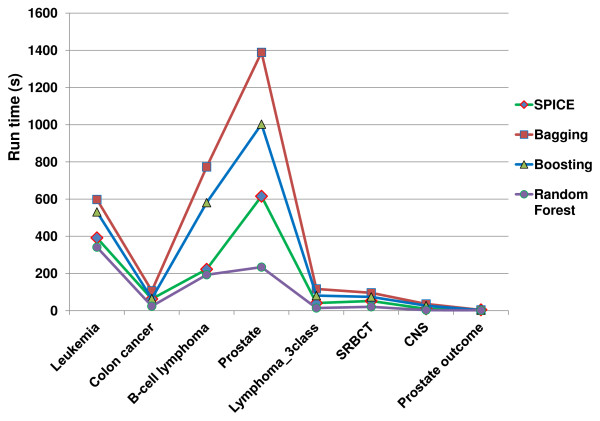
**The runtime of Spicecompared to other methods.**

Figure 5 shows the runtime of Spice and the benchmark methods on eight microarray datasets with 30 iterations as the stopping criterion. Our experiments were conducted on a PC with an Intel Core 2 Duo CPU (2.2GHz) and 6GB of RAM. All algorithms were implemented in the Matlab programming language.

For the eight datasets we tested, it shows that our Spice algorithm is much faster than bagging and boosting. While Spice is slower than random forest on some datasets, Spice could achieve better prediction accuracy on those datasets.

#### Generalization

Spice can be considered one of meta-learning ensemble algorithms [84], because Spice can employ an arbitrary base classifier. Table 7 shows its effectiveness compared to a single classifier using different base classifiers on the Colon cancer dataset with the 10-fold cross-validation. Spice improves the prediction accuracy of a single classifier, namely by about 30%, 14%, and 7% for Naïve Bayes, CART decision tree, and linear SVM, respectively. Thus, Spice can be applied to improve some base classifiers other than decision tree, which makes Spice more useful.

**Table 7:** Accuracy improvement over a single base classifier

**Classifier**	**Single classifier**	**Spice**
Decision Tree (CART)	0.73	0.87
Naïve Bayes	0.57	0.87
Linear SVM	0.82	0.89

## Conclusion

In this paper, we addressed the important and challenging problem of enumerating statistically significant and application-relevant component interplays that are key contributors to the system’s phenotype. We presented Spice, an effective, iterative feature subsets enumeration method that discriminates between different systems’ phenotypic states on both instance-based data and network-based data. Spice successfully identified cancer-related genes from various microarray data sets and found enzymes or COGs associated with biohydrogen production and motility phenotype by microbial organisms. Spice also improved the predictive skill of the system’s phenotype determination by up to 10% relative to individual classifiers and/or other ensemble methods, such as bagging, boosting, random forest, nearest shrunken centroid, and random forest variable selection method.

## Method

**Figure 6:**
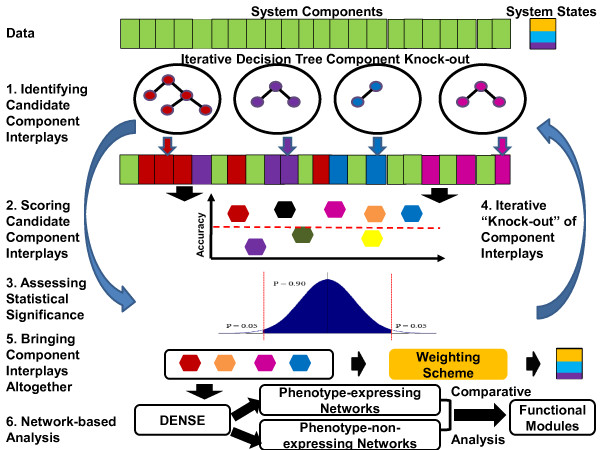
**The overview of S**pice**’s key steps.**

The key steps underlying Spice are shown in Figure 6. At a higher level, Spice first identifies a candidate component (feature) set (Step 1: identifying candidate component interplays section), it then scores its phenotype specificity-determining skill (Step 2: scoring candidate component interplays section) along with statistical significance assessment (Step 3: assessing statistical significance section). These three steps are repeated in an iterative fashion by “knocking out” the selected candidate component sets until the stopping criterion is met (Step 4: iterative “knock-out” of component interplays section). Finally, the ensemble of classifiers is formed to predict the system’s phenotype(s) given the values of all its component-interplay groups (Step 5: bringing component interplays altogether section). An additional step is added between Step 4 and Step 5 to ensure that the identified systems components are more strongly linked to the phenotype through comparative analysis of biological networks (Detecting biologically relevant component interplays through biological networks section). Next, we explain each of these steps in more detail.

### Step 1: identifying candidate component interplays

We hypothesize that if the component is key to defining the system’s phenotype, then its value distributions will be separable between the observations from different phenotypes. If the separation is strong, then such a component, alone, is likely able to discriminate system phenotypes. And almost any method, such as entropy-based, would likely succeed in detecting those components. However, with real data sets such a strong separation is less likely. Hence, one should strive for discovery of separation signals that while being weaker at the individual component level, they—as a group—should be able to discriminate between system phenotypes.

Therefore, the effective analysis should not only include an individual component with a strong discriminatory signal, but also extend to a group(s) of interplaying components out of a set of thousands of components. This creates a multiplicity of possible combinatorial interplays to search for and excludes a possibility for a brute-force enumeration. Thus, our goal is to provide a framework for automatic exploration of such combinatorial interplays that could offer both the computational efficiency and the application domain relevance.

To address this issue, we propose to employ the multi-level paradigm via divide-and-conquer strategy. The multilevel paradigm is known for its effectiveness when solving very large-scale scientific problems. In the context of linear systems of equations, for instance, algebraic multi-grid methods, have been devised to solve linear systems by essentially resorting to divide-and-conquer strategies that utilize the relationship between the mesh and the eigen-functions of the operator. In the data analysis field, however, methods that take advantage of the multi-level paradigm are less explored. A few recent studies include [85] as well as the top-down divisive clustering or spectral graph partitioning techniques.

Specifically, the intuition behind our approach stems from the well-known concept of modularity, introduced by Hartwell *et al.*[86], as a generic principle of complex system’s organization and function. These functionally associated modules often combine in a hierarchical manner into larger, less cohesive subsystems, thus revealing yet another essential design principle of system organization and function–hierarchical modularity. Thus, our method first identifies modules of system components with putatively stronger associations within the modules than between the modules. This process divides all system components into modules that likely function together to define what phenotypic state the system is in. The process further conquers each of these modules in order to refine the specificity of the inter-component relationships within the module.

**Figure 7:**
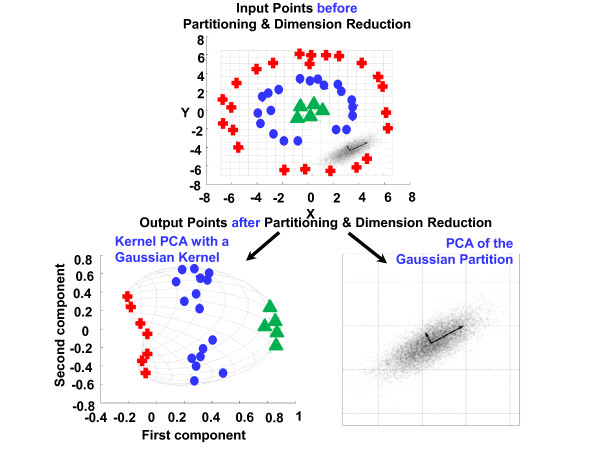
An illustration of divide-and-conquer strategy for multi-level dimension reduction.

Figure 7 shows an illustration of this divide-and-conquer approach to multilevel dimension reduction. The sample artificial input set shown contains two substructures: points from a multivariate Gaussian distribution (grey) and the three groups of colored points arranged into nested rings (top). (Note that the color of the points is only there to show how the data groups together before and after the partition followed by dimension reduction). The standard PCA result performed on the monolithic set is mediocre, i.e., distinguishing the four different groups is impossible using only linear PCA. After partitioning the set, the “appropriate” technique is applied to each partition (bottom): the kernel PCA to the nested ring points (left partition) and the linear PCA to the Gaussian cluster (right partition). As a result, not only is the size of the data reduced for each partition, but also the four groups become distinguishable using only the first principal component.

Unlike the example in Figure 7, in the context of our problem, we deploy decision tree-based procedure to divide the feature set into non-overlapping partitions and apply the “appropriate” classification technique to each partition. The reason is that due to highly underdetermined nature of our problem, subsampling of the input data sample could possibly lead to an unreliable inference methodology. Likewise, due to a possibly non-linear interplay between the system’s features, it would be more desirable to divide the system components into “blocks” with possibly stronger interconnects within the blocks and weaker inter-connects between the blocks. This strategy is inspired by the modularity principle of complex systems. Thus, a higher-level supervised separation of the high dimensional feature space into the rectangular shape hyperspaces is achieved via information-theory driven decision boundaries with a subsequent refinement of decision boundaries within the identified subspaces (see Step 2).

We propose a decision tree-based methodology for our feature space partitioning. The features in a decision tree are considered as one feature subset, and each feature is a system component. There are multiple reasons for why we choose decision tree based methodology, including (a) efficiency to process many features (unlike BBNs that are exponential in the number of features), (b) inherently multiclass by nature, and (c) the ability to handle continuous and multi-variate types of features (unlike NNs for which distance metrics are poorly defined for mixed data types), among others. We use the CART-decision tree algorithm [87] to select a set of discriminatory features from the available feature space. Basically, CART builds a decision tree by choosing the locally best discriminatory feature at each split step based on the Gini Index Impurity Function. To avoid overfitting, CART employs backward pruning to build smaller, more general decision trees. CART chooses features in a multivariate fashion, which allows the feature selection process to find a set of discriminatory features instead of considering one feature at a time.

More importantly, especially, in the context of underdetermined or unconstrained problems, CART’s inherent feature pruning capability often leads to a fewer number of components, or smaller size modules. This is a desirable property for building a more robust classifier downstream of our analysis pipeline (Step 2 and Step 5). Also, decision boundaries themselves could result in rules that are more interpretable and could provide additional insights to domain scientists on the magnitude of the feature attributes that affect a system’s phenotype. The reason is that not only is it important to know what group of features is contributing to the system’s phenotypic state but to what extent the feature values could change the system’s phenotypic state. For example, if the expression of a particular gene becomes above a certain threshold, then this causes a “knock-out” of a particular metabolic pathway. With decision trees, the full feature space gets partitioned into hypersubspaces by the decision rules of the form of _*ai*_≤_*fi*_≤_*bi*_. Once this high-level factors contributing to the system’s phenotype are learned, more complex (e.g., non-linear or conditional) relationships between the components in the group could be learned by more sophisticated classifiers, such as BBNs or kernel SVMs (see Step 2).

### Step 2: scoring candidate component interplays

Candidate system’s components identified in Step 1 are next assessed in terms of their collective ability to contribute to the system’s phenotypes. Basically, the goal is to define a scoring function that could measure how well this group of components (features) discriminates between system phenotypic states. On the one hand, mutual information (MI) for an individual component could be used with its proper generalization to a group of components. However, robust probability estimation—an essential step in MI definition—requires a large sample size, which is often unavailable for underdetermined systems. Moreover, the generalized MI is biased toward the presence of a component in the group with high information content.

Due to these limitations, we define a scoring function in terms of classification accuracy provided by multivariate discriminant methods, such as SVMs, BBNs, neural networks, or decision trees. Specifically, we ask a question: if only a candidate component set were used to determine the system’s phenotypic state, how much predictive skill this set could have. Since individual components within the candidate group could be related to each other in a complex manner, we first let a proper classifier (e.g., kernel SVM or BBN) learn this complex relationships from the entire group of features and choose the accuracy of the best performing classifier as the scoring measure of the putative components’ interplay (see Line 6–7 in Algorithm 2 of Additional file 8). Note that different candidate groups may require different classifiers—the best performing classifier model is chosen both for Step 3 and for Step 5. [For our experiments, we use training accuracy].

### Step 3: assessing statistical significance

Given a candidate feature set (Step 1) and its predictive skill score (Step 2), we next assess statistical significance of this score, namely, how likely a similar skill score could be observed at random. Specifically, we want to use the confidence level for the classification accuracy to sift phenotype-specificity determining component groups. It is expected that the statistically significant, highly scored component groups are application-significant. For example, a group of candidate genes could be biologically significant for biohydrogen production or cancer phenotype expression (see Phenotype-specificity determining components sections).

It is worth observing that, generally, sample instances within the same system phenotype tend to be more similar than those from the other phenotypes. Hence, separation of feature value distributions between the samples from different states will be relatively clearer, and thus classification accuracy—as a measure of feature set’s discriminatory power—can be biased. This implies that standard statistical testing like shuffling the phenotype (class) labels is not acceptable.

Thus, to provide a robust assessment of statistical significance, we measure an empirical *p*-value of each candidate feature set using the Monte Carlo procedure described in [88]. Specifically, for each feature subset, we randomly sample *N* feature subsets (*N*=1,000) from the entire feature set of the same size as our candidate set, and compute the corresponding accuracies of the classifiers built from these feature sets. Then, we estimate an empirical *p*-value of the target feature subset as *p*=(*R* + 1)/(*N* + 1), where *N* is the total number of random samples (*N*∼1,000) and *R* is the number of these samples that produce a test statistic greater than or equal to the value for the target feature subset. This corresponds to the percentile where our target score falls onto within the accuracy distribution for *N* samples. In our experiments, the selected *p*-value meets 95% confidence level. Please find the detailed pseudo-code for the statistical significance assessment in Additional file 8.

### Step 4: iterative “knock-out” of component interplays

The candidate component-interplay group identified in Steps 1-3 is probably not the only group of system components that is responsible for a system’s behavioral phenotypic state. For example, such a group of enzymes could contribute to a direct conversion of a particular type of sugar to ethanol, but there could still be other groups of genes required for ethanol production, such as regulators of these enzymes’ expression in the cell, transporters of different sugars from the environment into the cell, or stress response regulators that detect toxin (i.e., ethanol) concentration level in the cell. In addition, if a subsystem is critical for a specific system’s function, then it often gets replicated (e.g., multiple gene copy numbers in the genome) in the complex system; this redundancy contributes to system’s robustness. Therefore, our task is not simply to identify a single “best” group but, ideally, to enumerate them all.

The combinatorial nature of this task necessitates heuristic approaches. Our strategy is inspired by the way biologists often conduct their mutagenesis studies. Namely, they knock-out a group of genes (e.g., via gene deletion) and observe the *mutant* system’s response. By analogy, our methodology knocks-out the selected candidate feature sets and proceeds with Steps 1-3 on the mutant system in an iterative fashion until some stopping criterion is met (see Line 3 in Algorithm 2 of Additional file 8). Under this approach, each iteration produces a subset of features out of the current feature set (see Line 5 in Algorithm 2 of Additional file 8), then removes these features from the set so that they can’t be selected again (see Line 15 in Algorithm 2 of Additional file 8).

There are several different criteria that could be used to decide when to stop the iterative process. Ideally, one would observe a monotonically decreasing scoring value with the number of iterations and will stop once the score falls bellow a certain threshold. However, no theoretical grounds could be provided for such a monotonic behavior of the scoring function under the scenario of iterative feature set knock-outs. In fact, we empirically observed a fluctuating behavior of the scoring function with the number of iterations. Therefore, due to inherently high dimensional data, we set the threshold on the maximum number of iterations as our stopping criterion. Line 3–17 in Algorithm 2 of Additional file 8 summarizes the aforementioned iterative knock-out procedure.

### Step 5: bringing component interplays altogether

While the enumerated set of putative system’s component interplays is important in its own right (as illustrated in Results and discussion section), here we combine them altogether by building an ensemble of classifier models from Step 3. Thus, unlike traditional classification methods that aim to find the single subset of features that offer the most optimum classifier performance, our goal is to enumerate suboptimal feature sets that could provide insights on what factors and their inter-factor relationships could determine the specificity of the system’s phenotype. We then combine these subsystems through the framework of the ensemble methods in order to construct a system-level predictor of system’s behavioral states.

In the last step (Step 5 in Figure 6), we need to combine the predictions of all the classifiers that pass statistical significance criterion (Step 3) to come up with the final prediction value. In order for the ensemble to make a prediction, each classifier is given a weighted vote, and the class with the most votes is the prediction of the ensemble (see Line 18 in Algorithm 2 of Additional file 8). We tested three possible weighting schemes: a simple majority voting scheme, in which every classifier is given equal weight; a training accuracy-based method, in which every classifier is weighted based on its training accuracy; and an internal cross-validation-based voting, in which each classifier is weighted by that model’s cross-validation accuracy on the original training data.

Two of the key characteristics for building a robust classifier ensemble include (a) the diversity among the classifier models in the ensemble [84] and (b) the reasonably high accuracy of the individual members in the ensemble. In our case, the former is ensured due to our feature set knock-out strategy (Step 4) and the latter is guaranteed by a combination of decision-tree based feature enumeration (Step 1), the scoring function (Step 2), and the statistical significance assessment (Step 3) that, in combination, also reduce possible redundancy among the models and thus reduce the possible bias (e.g., due to a significantly large portion of highly similar models). By bringing the enumerated component interplays altogether (Step 5) a good ensemble of classifiers can be achieved (as illustrated in Results and discussion section).

### Detecting biologically relevant component interplays through biological networks

Thus far, we have presented how to detect component interplays from an instance-based data. And it has been shown that the system components enumerated by Spice often form functional modules or communities. However, an additional step could be added between Step 4 and Step 5 to ensure that the identified systems components are more strongly linked to the phenotype through biological networks.

The gene functional association networks used in this paper are obtained from the STRING database [89]. The nodes in the networks are genes. And a pair of nodes is connected with an edge if the corresponding genes are considered to be functionally associated by some evidence. The edge weights are assigned by the STRING database based on the evidence that support the functional association [89]. A threshold above 700 is considered as “high confidence” in the STRING database, so we only keep the edges with weights above 700.

After the network construction, we employ our Dense and Enriched Subgraph Enumeration (DENSE) algorithm [90] to enumerate “dense and enriched” subgraphs in each network. Intuitively, DENSE works as follows, given an organismal protein (gene) functional association network and a set of proteins (genes) as the query, DENSE enumerates all the dense subgraphs that are enriched by the query proteins. Every subgraph generated by DENSE contains at least *γ*percentage of nodes that are from the query protein set, and each node in the subgraph is adjacent to at least *μ*percentage of the other nodes in the subgraph. And in simple terms, the algorithm is able to extract the proteins that are functionally associated with the query proteins (i.e., form functional modules with them). In the paper [90], a biologist’s knowledge priors have been incorporated into the query set. Here, we use the phenotype-determining components generated by Spice as the query set for the DENSE algorithm. The default parameter values, *μ* =75 and *γ* =0.1, are used to find all highly connected (but not fully connected) subgraphs that contain at least one query node. [For more details on the DENSE algorithm and the software, please, refer to paper [90]].

The “dense enriched” subgraphs generated by DENSE are assumed to be the functional modules, because we start with the functional association network and impose the *μ*parameter to generate the highly connected subgraphs. However, a further functional enrichment analysis is performed on the discovered modules by using the GO TERM FINDER tool [91]. And the result shows that the discovered modules are indeed functionally coherent. [Since our work does not focus on the functional enrichment analysis, the experimental results are available upon request].

While DENSE is an effective and efficient algorithm to identify the functional modules in a biological network, it can only be applied to a single network at a time. However, we would like, using both phenotype-expressing and non-expressing organisms, to identify functional modules that are more biased towards the target phenotype. Thus, in this section, we propose an effective methodology to discover functional modules using DENSE but extending the procedure to utilize both phenotype-expressing and non-expressing organisms.

#### Definition 1 (*β***-Similar Dense Subgraphs**)

Given two dense subgraphs generated from two different networks, we call the two subgraphs *β*-similar dense subgraphs if they share at least *β*percentage of nodes corresponding to homologous genes.

For a set of networks corresponding to phenotype-expressing organisms, we hypothesize that the conserved *β*-similar dense subgraph (see Definition 1) across the group of networks are the phenotype-associated functional modules. After generating all “dense enriched” subgraphs from each biological network by DENSE, we first detect the *β*-similar dense subgraphs across two networks based on the Definition 1, and then check if the *β*-similar dense subgraphs detected in the previous two networks are conserved in the third network. This procedure is continued until all networks in the group are examined. Our algorithm may miss some of the phenotype-related modules if the stringent value of *β*=100 are used. Hence, we chose a *β*value of 75 (midpoint of 50 and 100) to identify highly conserved (but not identical) subgraphs across all networks as the most probable modules. Detection of the conserved *β*-similar dense subgraphs in a group of networks can also help us filter out some spurious query nodes (see Cancer-related genes section), which are generated by our Step 1-Step 4.

We can take it one step further and use a group of contrast biological networks (i.e., networks of organisms that do not express the phenotype) to filter and obtain dense subgraphs that are not only identified as conserved in the previous step but are also “biased” towards the target phenotype. Here, by biased, we mean occurring in phenotype expressing organisms but not occurring in the phenotype non-expressing organisms. To achieve this goal, first, the networks are partitioned into different groups according to the phenotype(s), and then the *β*-similar dense subgraph detection algorithm is applied to each group of networks. After getting all the conserved *β*-similar dense subgraphs from all groups, we remove all the common conserved *β*-similar dense subgraphs appearing in at least two groups of networks.

As noted, three parameters, *γ*, *μ* and *β*, are used in our algorithm. The thresholds of the parameters depend on the application. But because the computational time of DENSE algorithm is relatively small, users can try different thresholds and use their prior knowledge to design the query sets (e.g., pathway-phenotype associations) to validate the results. [The parameter sensitivity analysis is available upon request]. And similar to other comparative analysis methods, our results are sensitive to the phylogenetic diversity of the organisms we chosen. A scoring function based on the phylogenetic diversity could be considered as an option to address this problem.

Our work is different to other network-based identification methods in a number of ways: (1) we can discover dense, possibly overlapping subgraphs of a single network or groups of networks; and (2) we are able to identify “fuzzy functional modules” that are enriched by some target set of proteins (genes).

## Competing interests

The authors declare that they have no competing interests.

## Author’s contributions

ZC developed and implemented the computational model and the algorithm based on ideas suggested by NFS. ZC and KP conducted the computational experiments. YS, AR, ZC, and KS provided biological validation. ZC, KP, AR, and NFS provided the initial draft of the manuscript. ZC, KP and NFS provided the revised manuscript. JM suggested and supervised the study related to the hydrogen production. NFS provided the problem statement, supervised the development of the computational methodology, and provided suggestions on methodology validation. JM, KS, and NFS contributed to preparing the final version of the manuscript. All authors have read and approved the final manuscript.

## Supplementary Material

Additional file 1 **17*H ***^**2**^**-producing and 11 *H***_**2**_**-non-producing microorganisms.** Click here for file

Additional file 2 **Enzymes associated with biohydrogen production detected by Spice**. Click here for file

Additional file 3 COG data with 17 microorganisms for biohydrogen production. Click here for file

Additional file 4 **COG modules related to biohydrogen production detected by Spice**. Click here for file

Additional file 5 COG data with 141 microorganisms for motility phenotype. Click here for file

Additional file 6 **COG modules related to motility detected by Spice**. Click here for file

Additional file 7 Cancer-related gene modules detected on Leukemia data. Click here for file

Additional file 8 Pseudo-code of SPICE algorithm. Click here for file
